# Global burden and epidemiological determinants of pulmonary arterial hypertension among women aged 55 years and older: a GBD 2023 analysis

**DOI:** 10.3389/fmed.2026.1853948

**Published:** 2026-09-07

**Authors:** Guang-Wei Huang, Yue Cui, Zhi-Cheng Jing, Hai-Long Dai

**Affiliations:** 1 Department of Cardiology, Key Laboratory of Cardiovascular Disease of Yunnan Province, Clinical Medicine Center for Cardiovascular Disease of Yunnan Province, Yan’an Affiliated Hospital of Kunming Medical University, Kunming, China; 2 Department of Cardiovascular Medicine, Anshun City People's Hospital, Anshun, China; 3 Department of Cardiology, State Key Laboratory of Complex Severe and Rare Diseases, Peking Union Medical College Hospital, Chinese Academy of Medical Sciences and Peking Union Medical College, Beijing, China; 4 Department of Cardiology, Guangdong Cardiovascular Institute, Guangdong Provincial People's Hospital, Guangdong Academy of Medical Sciences, Southern Medical University, Guangzhou, China

**Keywords:** China, GBD 2023, global burden, pulmonary arterial hypertension, women aged 55 years and older

## Abstract

**Background:**

Pulmonary arterial hypertension (PAH) exhibits significant sex disparities; however, the impact on women aged ≥55 years—a population-level proxy for postmenopausal women who experience substantial hormonal and cardiometabolic changes—has not been comprehensively assessed. This research assessed long-term trends, regional variations, and structural factors influencing PAH in women aged ≥55 years.

**Method:**

This study utilizes standardized estimation data from GBD 2023, focusing on women aged 55 years and older as a population-level proxy for postmenopausal status. Joinpoint regression analysis was employed to investigate the trends in load. Decomposition analysis was utilized to measure the distinct impacts of population increase, aging, and epidemiological shifts. Frontier analysis was utilized to assess health output efficiency across different nations, while the ARIMA model was employed to forecast data until 2040.

**Results:**

In 2023, the Age-Standardized Incidence Rate (ASIR) showed a six-fold variation among nations (0.18–1.06 per 100,000), whilst the Age-Standardized Mortality Rate (ASMR) demonstrated almost a 160-fold disparity (0.007–1.13 per 100,000), with Central Asia and sub-Saharan Africa identified as high-burden hotspots. Between 1990 and 2023, global ASMR decreased by over 40%, while ASDR diminished by almost 35%. In persons aged 55 years and older, the age-specific incidence rate (ASIR) and age-specific prevalence rate (ASPR) for females were 1.3 to 1.8 times greater than those for males. Population growth accounted for 40–65% of the increases in burden in South Asia, East/Southeast Asia, and Africa, while aging was the predominant factor in China and high-SDI regions. By 2040, global mortality and disability-adjusted life years (DALYs) are anticipated to persist in their decline, whereas incidence and prevalence are likely to stabilize; China’s age-standardized incidence rate (ASIR) is forecasted to maintain a high plateau, while age-standardized mortality rate (ASMR) and age-standardized DALY rate (ASDR) will continue to diminish, exhibiting a steady trajectory.

**Conclusion:**

This study reveals significant and enduring geographical disparities in the prevalence of PAH among women aged 55 years and older worldwide. Women aged 55–69 years represented the age group with the highest population-level incidence of PAH in the GBD estimates and may therefore warrant priority in surveillance and preventive strategies. High and middle SDI countries must swiftly establish long-term monitoring and chronic management systems specifically designed for women aged ≥55 years. Targeted policies and healthcare services are crucial for mitigating the global impact of PAH.

## Introduction

Pulmonary hypertension (PH) is a progressive cardiovascular condition marked by sustained increases in pulmonary vascular resistance and pulmonary arterial pressure, resulting in right ventricular failure and early mortality (1–3). The global burden has consistently increased in recent decades, especially in resource-constrained environments where delayed diagnosis and restricted treatment access worsen outcomes. Despite the expansion of epidemiological research, significant variation in the incidence of PH among populations remains inadequately comprehended, and women aged ≥55 years—one of the most vulnerable yet underexplored subgroups—have garnered insufficient targeted attention. PH is an intricate concept that includes several causes and classifications, with unique varieties exhibiting evident differences in their pathogenic mechanisms and illness course (4). In the GBD 2023 framework, PAH is identified using ICD-based coding algorithms that may partially overlap with broader PH categories. Therefore, the disease entity analyzed in this study may not correspond exactly to clinical WHO Group 1 PAH defined by right-heart catheterization criteria (5, 6). In comparison to other pulmonary hypertension groups, PAH demonstrates a pronounced biological sensitivity to sex, age, and endocrine factors. Women aged ≥55 years constitute a distinct at-risk demographic owing to sudden decreases in estrogen levels, diminished vasoprotective signaling, increased inflammation and oxidative stress, and a greater incidence of metabolic syndrome. These physiologic shifts may jointly enhance vulnerability to pulmonary vascular remodeling.

Recent study has emphasized the significant influence of the interaction between gender and age on the susceptibility to and progression of PAH. women aged ≥55 years, owing to a significant reduction in estrogen levels (7), diminished vascular protective effects (8), heightened oxidative stress and inflammation (9), and a high prevalence of metabolic syndrome, are typically seen as more susceptible to pulmonary vascular remodeling (10). Clinical observations and fundamental research indicate that estrogen receptor signaling, immune-inflammatory control, metabolic pathways, and right ventricular adaption display distinct patterns in women, particularly women aged ≥55 years, potentially influencing disease development significantly. In PAH, gender disparities are notably pronounced. Despite the increased occurrence of PAH in women relative to men (11), women generally demonstrate superior RV function and a more favorable prognosis, a phenomenon referred to as the ‘estrogen paradox’ (12). Recent scientific research has revealed that estrogen and its metabolites have bidirectional effects on pulmonary vasculature and right heart function (13). These effects are both time-dependent and organ-specific: from one perspective, estrogen has the potential to stimulate the growth of pulmonary vascular smooth muscle (14); from another perspective, estrogen receptor signaling helps improve the adaptability of the RV and optimize right heart function (15). Comprehending this intricate hormonal regulatory process may yield novel therapeutic targets for ameliorating pulmonary vascular diseases and enhancing right ventricular performance.

With the rapid acceleration of global population aging, women aged ≥55 years are anticipated to emerge as one of the most rapidly expanding demographics impacted by PAH. Nevertheless, the majority of contemporary studies depend on single-center or region-specific cohorts, which lack cross-national comparability and integration of socioeconomic context. As a result, the geographical variability, temporal patterns, and structural factors influencing PAH load in women aged ≥55 years are little characterized. The GBD 2023 dataset offers a distinctive chance to rectify these deficiencies through standardized and globally consistent estimations.

This study methodically examines the burden of PAH in women aged 55 years and older at global, regional, SDI-based, and national levels. Through Joinpoint trend analysis, spatial pattern characterization, inequality assessment, decomposition modeling, hierarchical clustering, frontier analysis, and ARIMA forecasting, we sought to: (1) quantify geographic and SDI-related disparities in PAH burden; (2) identify principal epidemiological drivers affecting regional variations; (3) elucidate the unique epidemiological status of women aged ≥55 years within the broader PH spectrum; and (4) provide evidence to facilitate targeted prevention, resource allocation, and policy formulation across different health-system contexts.

## Methods

All data examined in this study is derived from GBD 2023, encompassing prevalence, disease severity, and mortality rates. These markers collectively offer a thorough evaluation of the disease burden. All such data is readily available on the Global Health Data website. In 2021, PAH was incorporated into the ‘Benchmark Chart’ as an independent condition, featuring comparable and consistent estimates of the PAH burden from 1990–2023. The contemporary hemodynamic criteria for PAH stipulate a resting mean pulmonary arterial pressure beyond 20 mm Hg, a pulmonary capillary wedge pressure below 15 mm Hg (16), and a minimum of 2 Wood units of pulmonary vascular resistance assessed via right cardiac catheterization (17). Echocardiography assesses pulmonary arterial pressure through tricuspid regurgitation velocity, frequently utilized in resource-constrained environments, potentially influencing diagnostic sensitivity and specificity (18). The GBD case definition necessitates a diagnosis of PAH corroborated by right cardiac catheterization or echocardiographic evidence. This study delineates the methodologies employed to assess the health burden of PAH in women aged ≥55 years. The age threshold of 55 years and above was employed to estimate the postmenopausal condition, consistent with prior epidemiological research utilizing analogous data sources (19). Because the GBD 2023 database does not provide individual-level information regarding menopausal status or age at menopause, women aged ≥55 years were used as a population-level epidemiological proxy for the postmenopausal population. This threshold was selected as a conservative cutoff above the average natural menopausal age reported in most populations worldwide, thereby minimizing the likelihood of including premenopausal individuals. Similar age-based definitions have been adopted in previous GBD-based epidemiological studies involving postmenopausal populations. Nevertheless, the timing of menopause varies across ethnic, geographic, and socioeconomic settings; therefore, this operational definition may not fully capture biologically confirmed menopausal status in all regions. Accordingly, the findings should be interpreted as reflecting the burden of pulmonary arterial hypertension among women of postmenopausal age rather than strictly confirmed menopause. Men aged ≥55 years were included as an age-matched comparator group to evaluate sex-specific differences in disease burden patterns. In addition, the disease category analyzed in this study was derived from the GBD 2023 database using ICD-based modeling strategies rather than hemodynamic confirmation criteria for clinically diagnosed WHO Group 1 pulmonary arterial hypertension (PAH). Although the GBD framework classifies this condition as PAH, the database does not provide sufficient clinical granularity to reliably distinguish isolated WHO Group 1 PAH from other pulmonary hypertension subtypes in all cases. Consequently, the estimated disease burden may partially reflect a broader spectrum of pulmonary hypertension phenotypes beyond catheter-confirmed PAH.

The global and regional social demographic profile, SDI, and regional classifications in ‘GBD 2023’ were used to examine alterations in open access burden. SDI classifies 204 countries and areas into five quintiles—high, upper-middle, middle, lower-middle, and low—according to socioeconomic and demographic criteria, offering a uniform framework for the comparison of health outcomes. ‘GBD 2023’ categorizes these countries and regions into 21 distinct areas for regional examination, based on geographic closeness and cultural affinity. This amalgamation of SDI and regional classifications offers a sophisticated framework for examining and elucidating the global distribution of open access burden, underscoring differences linked to socioeconomic and geographical determinants. Additional information regarding SDI and regional definitions is available in earlier publications.

Although previous experimental and clinical studies have proposed several endocrine and vascular mechanisms underlying sex differences in PAH, the present study is based on population-level GBD estimates. Therefore, our analyses are intended to describe epidemiological patterns and population-level associations rather than to evaluate individual-level biological mechanisms or establish causal relationships.

### Joinpoint trend analysis

Joinpoint regression analysis was performed using Joinpoint Regression Program version 5.0.2 (20) to evaluate temporal trends in age-standardized burden indicators from 1990–2023. Log-transformed age-standardized rates were modeled assuming constant variance. A maximum of three joinpoints was permitted, and the optimal number of inflection points was determined using Monte Carlo permutation tests. Annual percentage changes (APCs) and average annual percentage changes (AAPCs) with corresponding 95% confidence intervals (CIs) were calculated to quantify temporal trends. Trends were considered statistically significant when the two-sided *p* value was <0.05.

### Analysis of inequality

Health inequality was assessed using the Concentration Index (CI) and Slope Index of Inequality (SII) across SDI regions. CI was used to evaluate the relative distribution of disease burden according to socioeconomic development levels, whereas SII quantified absolute inequality gradients. Linear regression analysis was additionally applied to evaluate temporal changes in inequality indicators between 1990 and 2023 (21). The Concentration Index (CI) and Slope Index of Inequality (SII) were calculated using the healthequal package in R (version 4.3.2).

### Hierarchical clustering

Hierarchical clustering analysis was performed based on regional EAPC values for mortality and DALYs. Euclidean distance matrices were constructed, and Ward’s minimum variance method (Ward.D2) was applied to minimize within-cluster heterogeneity. Cluster assignments were determined according to dendrogram structure and relative cluster stability (22).

### ARIMA time-series forecasting analysis

Time-series forecasting was conducted using autoregressive integrated moving average (ARIMA) models following the Box–Jenkins methodology. Stationarity of the time series was evaluated using differencing procedures and autocorrelation diagnostics. Model parameters (p, d, q) were selected based on the Akaike Information Criterion (AIC) and Bayesian Information Criterion (BIC). Residual diagnostics were assessed using autocorrelation function (ACF), partial autocorrelation function (PACF), and Ljung–Box tests to confirm white-noise residuals. Forecasts were generated for the period 2024–2040 with corresponding 95% CI (23). To assess the predictive reliability of the ARIMA model, we performed backtesting as recommended. A summary of key validation metrics—including mean absolute percentage error (MAPE), root mean square error (RMSE), and 95% prediction interval coverage rate—and a detailed year-by-year comparison between predicted values and actual GBD 2023 estimates are available in the Supplementary Supplementary materials. A visual summary of the backtesting results is also provided in the Supplementary Supplementary Figure S1.

### Analysis of the correlation with the SDI

Conduct a correlation and regression study between the disease burden across various locations and the SDI to investigate the influence of social development levels on variations in the burden of PAH. Utilize linear regression and local polynomial smoothing (LOESS) curves to assess the distribution of markers along the SDI gradient, pinpoint locations with notable deviations in high burden regions, and elucidate the potential influence of developmental inequality on illness progression.

### Decomposition analysis

Decomposition analysis was conducted according to the standard GBD analytical framework to quantify the independent contributions of population growth, population aging, and epidemiological change to variations in incidence, prevalence, mortality, and DALYs between 1990 and 2023. The total absolute change in disease burden was partitioned into three additive components: Total change = population growth effect + population aging effect + epidemiological effect. Population growth effect represented changes attributable to increases in population size, aging effect represented shifts in age structure, and epidemiological effect reflected changes in age-specific disease rates after adjusting for demographic changes.

### Frontier analysis

Frontier analysis was used to evaluate health-system performance relative to socioeconomic development. The relationship between SDI and age-standardized mortality or DALY rates was modeled using nonparametric frontier estimation. The efficiency frontier represented the lowest observed burden achievable at a given SDI level. Effective difference was calculated as the vertical distance between each country and the frontier curve, with larger distances indicating poorer-than-expected health outcomes relative to development level. Frontier analysis was performed using a non-parametric Data Envelopment Analysis (DEA) approach implemented in R (version 4.3.2). The frontier curves were visualized using the ggplot2 package. Bootstrap resampling was not performed.

### Statistical analysis

All statistical analyses and visualizations were performed using R software version 4.3.3 (R Foundation for Statistical Computing, Vienna, Austria). Joinpoint analyses were conducted using Joinpoint Regression Program version 5.0.2. A two-sided *p* value <0.05 was considered statistically significant.

## Results

### Global spatial distribution of PAH burden indicators among women aged ≥55 years

**Figure 1:**
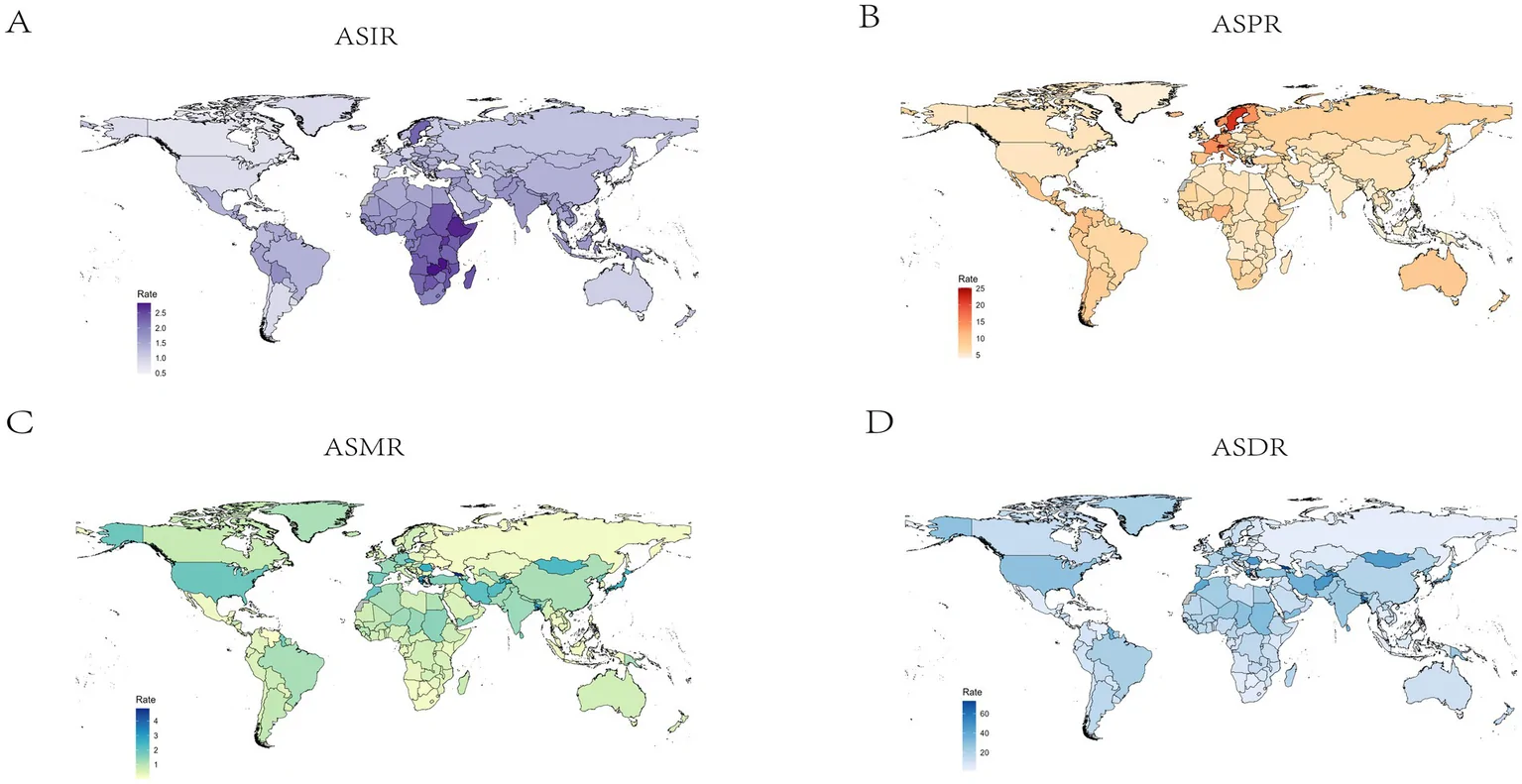
ASIR **(A)**, ASPR **(B)**, ASMR **(C)**, and ASDR **(D)** due to PAH in different countries in global in 2023. ASIR, Age-standardized incidence; ASPR, Age-standardized prevalence; ASMR, Age-standardized mortality rate; DALYs, Disability-adjusted life years; ASDR, Age-standardized DALY rate.

Global spatial distribution of PAH burden markers among women aged ≥55 years Worldwide, all four age-standardized metrics exhibit considerable geographical disparities (Figure 1; Supplementary Table S1). The distribution range of ASIR is roughly 0.18–1.06, with the greatest values predominantly located in Central and Eastern Sub-Saharan Africa, namely in countries such as Zambia, Ethiopia, Uganda, Rwanda, and Malawi. Concurrently, Western Europe, North America, and several East Asian regions, including Ireland, Canada, the United Kingdom, Portugal, and South Korea, exhibit the lowest levels globally. The global disparity in ASPR is significant, varying from around 1.32 to 7.12. High disease burden is primarily observed in high-income nations in Northern and Eastern Europe, creating a distinct high-value cluster that includes Switzerland, Sweden, the Netherlands, Israel, and Cyprus; conversely, countries such as Greenland, Pakistan, Afghanistan, Vanuatu, and Myanmar exhibit comparatively low ASPR. In general, numerous nations in Asia and Oceania exhibit a comparatively reduced disease burden. The most significant cross-national variances in ASMR range from 0.007 to 1.13. Central Asia and several sub-Saharan African nations, including Georgia, Tajikistan, Mongolia, Bermuda, and Niger, exhibit the highest mortality rates, whilst the Republic of Moldova, Estonia, Slovenia, Belarus, and Antigua and Barbuda report the lowest age-standardized mortality rates (ASMR). The spatial distribution pattern of ASDR largely aligns with ASMR, varying from around 0.59 to 31.12. The most significant burdens are concentrated in Central Asian nations, including Tajikistan and Georgia, and extend to areas such as Afghanistan, Niger, and Haiti; conversely, the lowest ASDR are typically observed in Central and Eastern European countries, such as the Republic of Moldova, Slovenia, Estonia, Croatia, and Belarus. Both ASMR and ASDR exhibit a significant burden corridor extending from Central Asia to sub-Saharan Africa, although numerous high-income regions sustain lower levels.

### SDI-related patterns of PAH burden among women aged ≥55 years across global regions and China

**Figure 2:**
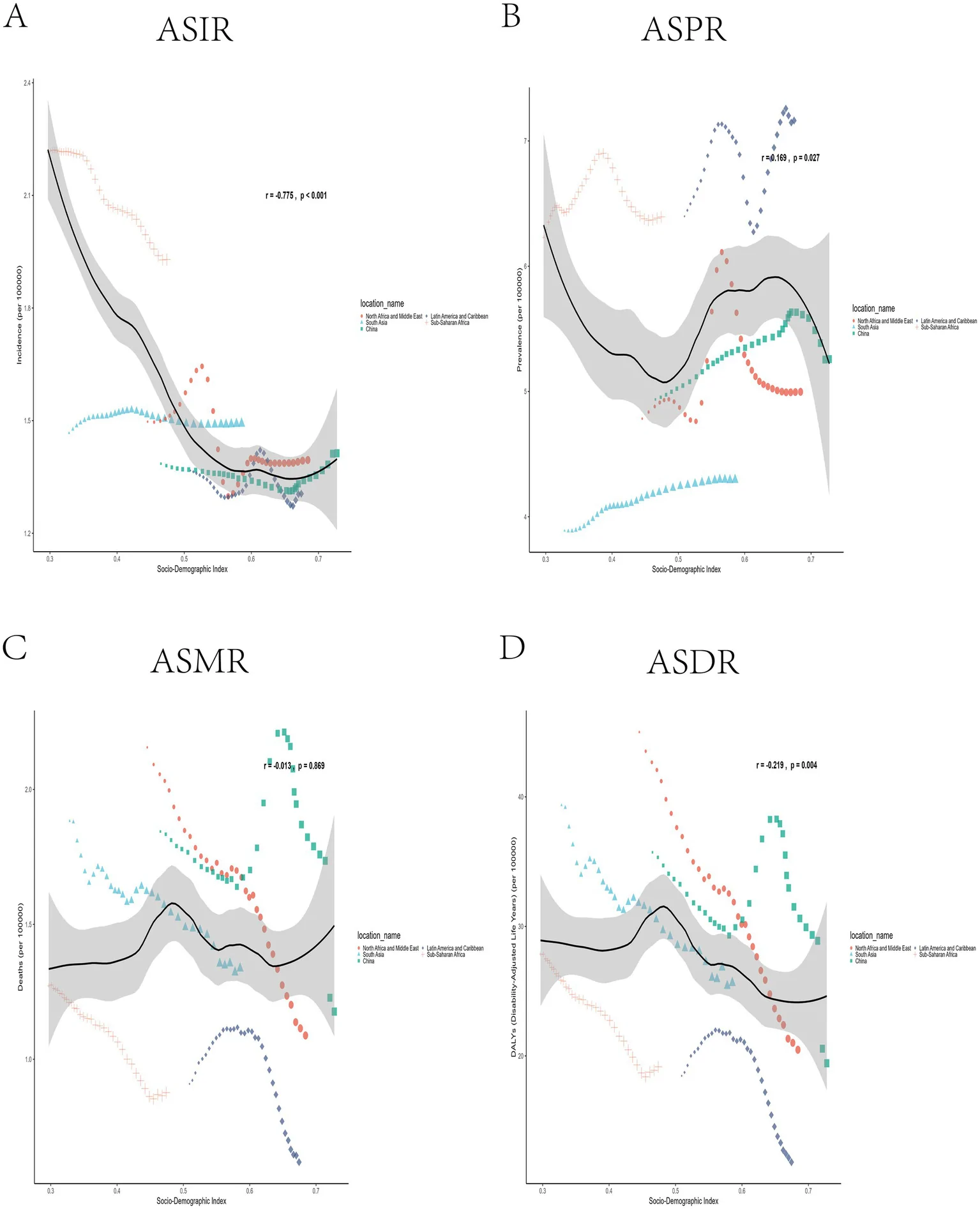
Association between SDI and the burden of PAH. **(A)** SDI vs. ASIR; **(B)** SDI vs. ASPR; **(C)** SDI vs. ASMR; **(D)** SDI vs. ASDR. LOESS curves with 95% CIs are shown; each dot represents a region or country. SDI, Socio-demographic Index; ASIR, age-standardized incidence rate; ASPR, age-standardized prevalence rate; ASMR, age-standardized mortality rate; ASDR, age-standardized DALY rate; CI, confidence interval.

Data from women aged ≥55 years throughout the seven major regions and China reveal considerable regional disparities in all four age-standardized measures, which vary according to the SDI. Figure 2A illustrates a significant decline in the ASIR with an increase in the SDI (*r* = −0.775, *p* < 0.001). Regions with low SDI, predominantly sub-Saharan Africa, exhibit elevated incidence rate intervals, whereas South Asia, East Asia, and China fall within the medium to medium-high SDI range, correlating with reduced incidence levels. In Figure 2B, ASPR exhibits a modest positive connection with SDI (*r* = 0.169, *p* = 0.027). The prevalence is broadly dispersed throughout various SDI ranges, with generally elevated disease levels in low SDI regions, whereas East Asia, Latin America, and China predominantly fall within the middle-high SDI range, correlating with moderate prevalence levels. Figure 2C demonstrates that the linear association between ASMR and SDI is not statistically significant (*r* = −0.013, *p* = 0.869). Countries with low Social Development Index (SDI) are predominantly found in the higher mortality rate spectrum, while regions such as South Asia, the Middle East–North Africa, and East Asia exhibit a distribution within the middle to upper-middle SDI range, with China’s mortality rate positioned in the mid-range among East Asian nations. Figure 2D illustrates a notable declining trend in ASDR as SDI rises (*r* = −0.219, *p* = 0.004). The burden level is greatest in low SDI regions, particularly Sub-Saharan Africa, whereas South Asia and the Middle East–North Africa exhibit a broader spectrum of values within the middle SDI range. East Asia and China are categorized in the medium-high SDI range, correlating with a lower to moderate disability load.

### Temporal trends of PAH across super regions, SDI levels, and China

**Figure 3:**
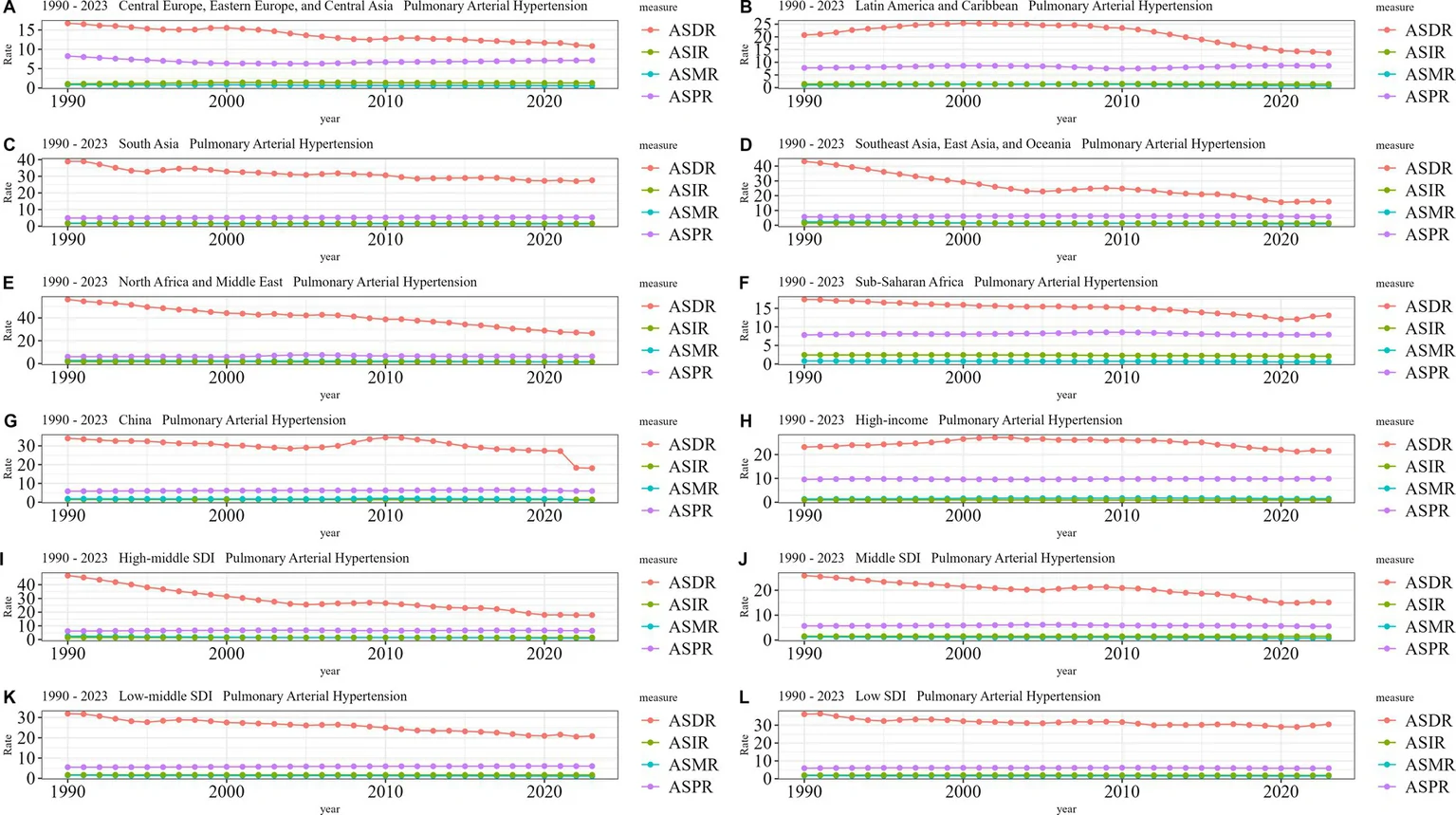
Age-standardized trends in PAH among women aged ≥55 years across seven global regions, SDI levels, and China, 1990–2023. **(A–L)** show the ASIR, ASPR, ASMR, and ASDR of PAH from 1990–2023 in Central Europe–Eastern Europe–Central Asia, Latin America and the Caribbean, South Asia, Southeast Asia–East Asia–Oceania, North Africa and the Middle East, Sub-Saharan Africa, China, High Income, and four SDI levels. Colored lines denote the four age-standardized indicators for each location. ASIR, age-standardized incidence rate; ASPR, age-standardized prevalence rate; ASMR, age-standardized mortality rate; ASDR, age-standardized DALY rate; SDI, sociodemographic index.

Between 1990 and 2023, temporal trends in PAH among older women displayed considerable geographical and Socio-Demographic Index-related variability (Figure 3). Central Europe, Eastern Europe, Central Asia, Latin America and the Caribbean, Southeast Asia, East Asia, Oceania, the Middle East, North Africa, and high-income regions all experienced a consistent decline in ASDR, although ASIR, ASMR, and ASPR remained low and stable. Conversely, South Asia and sub-Saharan Africa have consistently experienced greater pressures. ASDR in South Asia experienced a little decline before stabilizing, whilst ASPR in sub-Saharan Africa remained elevated, with minimal variation in the other indices. China’s ASDR has consistently declined since 1990, while its ASIR, ASPR, and ASMR have remained unchanged. Countries with high-medium and moderate SDI experienced significant declines in ASDR across all SDI categories, although other indicators remained unchanged. In the early 1990s, nations with low-middle and low SDI exhibited elevated ASDR, which subsequently declined over time. ASIR, ASPR, and ASMR were consistently low and constant. Generally, there were extensive declines in disability and mortality rates; nevertheless, areas with significant burdens experienced slower progress and ongoing rises across numerous indices.

### Comparison of the PAH burden in gender aged ≥55 years

**Figure 4:**
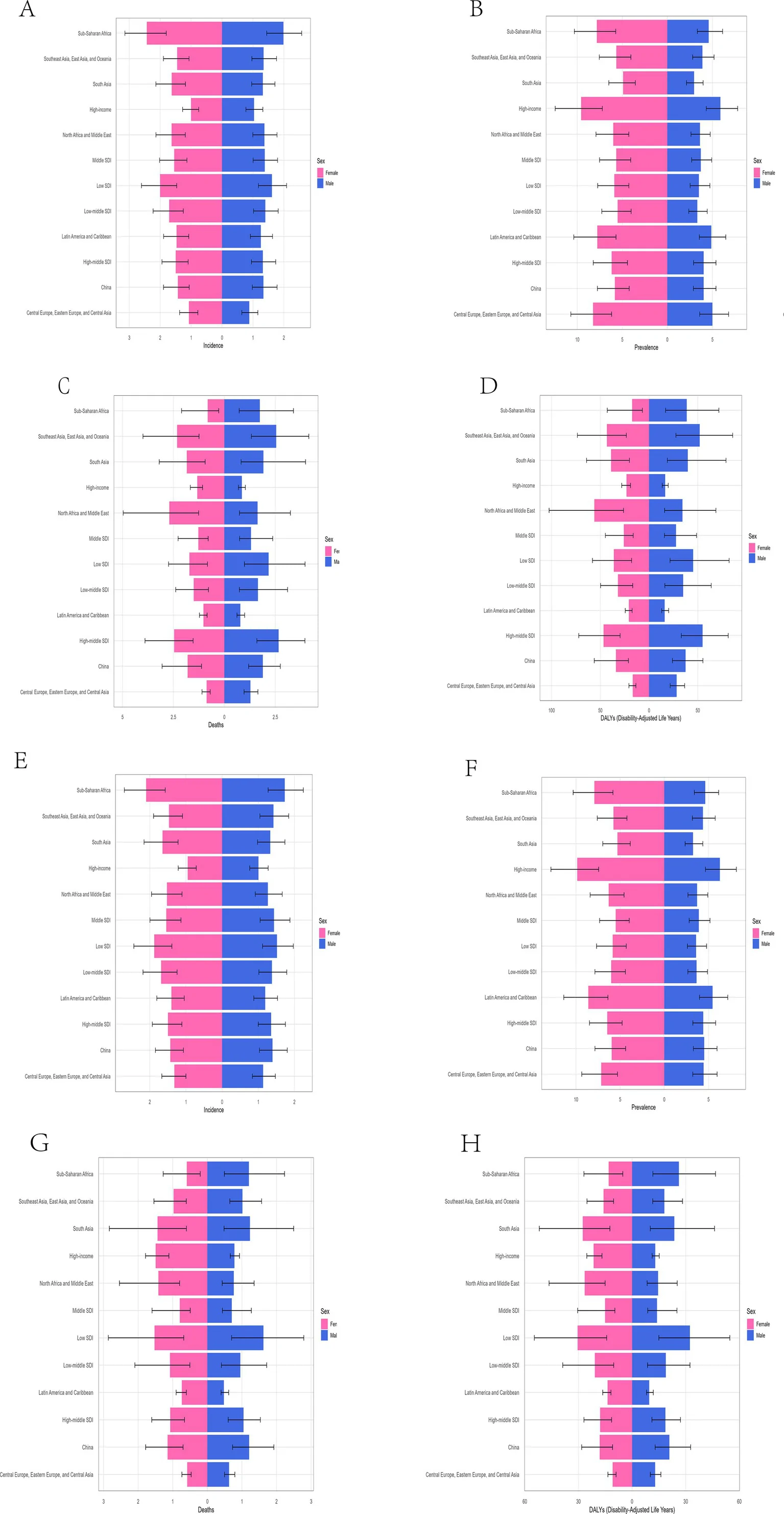
Sex-specific comparisons of age-standardized incidence rates [ASIR; **(A,E)**], prevalence rates [ASPR; **(B,F)**], mortality rates [ASMR; **(C,G)**], and disability-adjusted life-year rates [ASDR; **(D,H)**] of PAH among adults aged ≥55 years across global regions and SDI levels and China in 1990 and 2023. Pink bars represent females and blue bars represent males. Error bars indicate the 95% uncertainty intervals. ASIR, age-standardized incidence rate; ASPR, age-standardized prevalence rate; ASMR, age-standardized mortality rate; ASDR, age-standardized DALY rate.

From 1990–2023, the four age-standardized indices of PAH in the ≥55 age demographic exhibited consistent disparities in gender, geography, and temporal factors. ASIR and ASPR consistently demonstrated elevated rates in females compared to males across all years and the majority of regions, with this trend being especially pronounced in Sub-Saharan Africa, South Asia, Southeast Asia–East Asia–Oceania, and China; gender disparities were comparatively minimal in high-income regions and high middle SDI countries (Figure 4). Conversely, ASMR and ASDR had marginally elevated values in males relative to females, with discrepancies less pronounced than those observed for incidence and prevalence. Regionally, Sub-Saharan Africa consistently exhibited the highest levels across all four variables, succeeded by South Asia and Southeast Asia–East Asia–Oceania; conversely, high-income regions, high middle SDI regions, and China consistently recorded the lowest levels. From 1990–2023, the ASIR, ASPR, ASMR, and ASDR exhibited a general decline across most regions, especially in high and mid-high SDI regions, as well as in China, where the reduction was particularly significant. Conversely, the decrease was less pronounced in low SDI regions, which continued to bear the highest overall burden.

### Analysis of regional differences and age patterns worldwide

**Figure 5:**
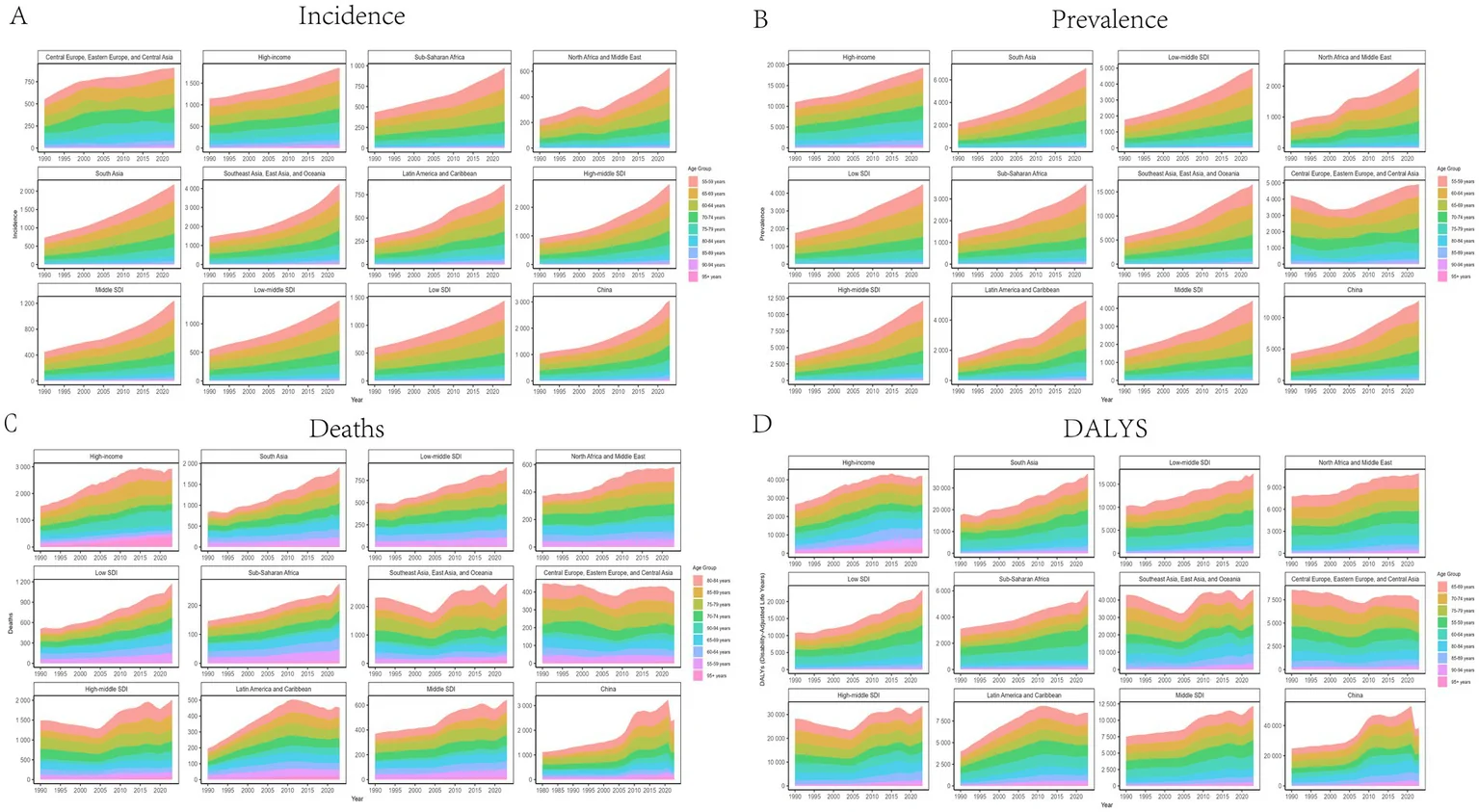
Regional and age-specific patterns of PAH burden among women aged ≥55 years, 1990–2023. **(A–D)** Temporal trends in incidence **(A)** prevalence **(B)** deaths **(C)** and DALYs **(D)** across global regions from 1990–2023. The right-side panels present the age-specific distribution of the four burden indicators in 2023, showing the relative contribution of each age group across regions.

Significant geographic variation in the prevalence of PAH among older women was noted at the regional level from 1990–2023 (Figure 1; Table 1; Supplementary Table S2). South Asia, sub-Saharan Africa, and low-SDI regions had the most significant and sustained rises in incidence and prevalence, while high-income countries like North America and Western Europe sustained continuously lower levels with relatively minor fluctuations (Figures 5A,B). The Middle East–North Africa, Latin America, and middle- to high-SDI nations had intermediate burden levels with moderate rising trajectories. China demonstrated consistent growth across all four metrics, positioning its load within the moderate-to-high spectrum, akin to trends noted in East Asia (Figures 5C,D). Age-specific studies revealed a uniform gradient across markers. The incidence and prevalence reached their zenith at ages 55–59, subsequently decreasing in new instances with increasing age, despite a continual rise in the overall number of affected individuals. DALYs peaked at ages 65–69, indicating the phase of maximum functional impairment. Mortality rates reached their zenith at ages 80–84, signifying that lethal outcomes were predominantly observed in the oldest demographic cohorts. The burden of PAH among older women exhibited significant geographical variability and a distinct age-stratified pattern, with the highest incidence occurring in early postmenopause, maximal disability load in late midlife, and mortality mostly in advanced age.

**Table 1:** Four age-standardized indicators of PAH in females aged 55 and above across different regions.

Location	Measure	Number 1990	ASR 1990	Number 2023	ASR 2023	EAPC (95% CI)
Central Europe, Eastern Europe, and Central Asia	Incidence	551.5 (402.4, 705.8)	1.1 (0.8, 1.4)	909.1 (689.6, 1149.9)	1.3 (1, 1.7)	0.3 (0.0,0.6)
	Prevalence	4238.9 (3179.4, 5507.7)	8.2 (6.2, 10.7)	4926.2 (3649.7, 6,440)	7.1 (5.3, 9.3)	−0.2 (−0.4,0.1)
	DALYs	8600.4 (6931.1, 10631.3)	16.7 (13.5, 20.7)	7470.4 (6,168, 9327.8)	10.8 (9, 13.5)	−1.2 (−1.3,−1.1)
	Death	448.2 (355.9, 564)	0.9 (0.7, 1.1)	402.2 (321.5, 506.7)	0.6 (0.5, 0.7)	−1.1 (−1.2,−1.0)
Latin America and Caribbean	Incidence	281.7 (206.3, 362.1)	1.5 (1.1, 1.9)	864.8 (645.7, 1,114)	1.4 (1, 1.8)	−0.1 (−0.2,0.1)
	Prevalence	1490.6 (1091.4, 1990.1)	7.8 (5.7, 10.4)	5305.6 (3934.6, 7019.5)	8.6 (6.4, 11.4)	0.1 (−0.1,0.3)
	DALYs	3969.5 (3330.9, 4674.6)	20.7 (17.4, 24.4)	8450.5 (7,295, 10137.8)	13.7 (11.8, 16.4)	−1.5 (−1.9,−1.0)
	Death	196.3 (162.4, 234.2)	1 (0.8, 1.2)	454.3 (374, 556.2)	0.7 (0.6, 0.9)	−1.1 (−1.6,−0.6)
North Africa and Middle East	Incidence	225.3 (164.3, 295.7)	1.6 (1.2, 2.1)	629 (457.3, 804.8)	1.5 (1.1, 2)	−0.4 (−0.6,−0.2)
	Prevalence	830 (591.4, 1095.3)	6 (4.3, 7.9)	2595.9 (1879.1, 3462.4)	6.3 (4.6, 8.4)	0.1 (−0.1,0.4)
	DALYs	7,768 (3616.5, 14173.9)	56.2 (26.2, 102.5)	10924.4 (6218.5, 19151.9)	26.5 (15.1, 46.5)	−2.1 (−2.2,−1.9)
	Death	374.1 (174.5, 686.7)	2.7 (1.3, 5)	582.6 (330.6, 1047.7)	1.4 (0.8, 2.5)	−1.7 (−1.9,−1.5)
South Asia	Incidence	734.5 (533.9, 965.4)	1.6 (1.2, 2.1)	2195.7 (1622.2, 2876.9)	1.6 (1.2, 2.2)	−0.0 (−0.1,0.0)
	Prevalence	2213.7 (1,601, 2931.9)	4.9 (3.6, 6.5)	7064.4 (5138.7, 9285.7)	5.3 (3.9, 7)	0.3 (0.3,0.3)
	DALYs	17562.1 (9081.5, 28904.5)	38.9 (20.1, 64.1)	36,762 (16529.5, 69166.3)	27.6 (12.4, 51.9)	−0.9 (−1.0,−0.8)
	Death	831.5 (424.3, 1441.9)	1.8 (0.9, 3.2)	1915.7 (804.4, 3772.5)	1.4 (0.6, 2.8)	−0.6 (−0.7,−0.5)
Southeast Asia, East Asia, and Oceania	Incidence	1444.3 (1061.2, 1885.3)	1.5 (1.1, 1.9)	4236.1 (3157.7, 5472.4)	1.5 (1.1, 1.9)	−0.1 (−0.2,−0.0)
	Prevalence	5631.9 (4022.5, 7510.8)	5.7 (4, 7.6)	16606.2 (12208.8, 21888.1)	5.8 (4.2, 7.6)	0.1 (0.0,0.2)
	DALYs	42986.5 (23180.2, 73120.3)	43.2 (23.3, 73.5)	46039.9 (29842.1, 72834.2)	16 (10.3, 25.2)	−2.8 (−3.1,−2.6)
	Death	2306.3 (1236.5, 3972.9)	2.3 (1.2, 4)	2816.7 (1754.4, 4452.4)	1 (0.6, 1.5)	−2.5 (−2.7,−2.2)
Sub-Saharan Africa	Incidence	434.7 (322.1, 561.2)	2.4 (1.8, 3.1)	976.9 (732, 1257.5)	2.1 (1.6, 2.7)	−0.5 (−0.6,−0.4)
	Prevalence	1397.5 (1022.8, 1842.1)	7.8 (5.7, 10.3)	3,681 (2704.5, 4798.4)	7.9 (5.8, 10.3)	−0.0 (−0.1,0.1)
	DALYs	3109.6 (1221.6, 7650.6)	17.4 (6.8, 42.8)	6094.8 (2420.8, 12540.5)	13.1 (5.2, 27)	−1.0 (−1.1,−0.9)
	Death	145.7 (49.3, 375)	0.8 (0.3, 2.1)	276.3 (95.6, 593.6)	0.6 (0.2, 1.3)	−1.1 (−1.2,−1.0)
High-income	Incidence	1147.4 (864.9, 1462.1)	1 (0.8, 1.3)	1860.5 (1,399, 2,376)	1 (0.7, 1.2)	−0.2 (−0.3,−0.2)
	Prevalence	10971.8 (8267.9, 14263.4)	9.6 (7.2, 12.4)	19,293 (14561.9, 25135.6)	9.9 (7.4, 12.8)	0.1 (0.0,0.1)
	DALYs	26553.7 (21827.5, 32164.4)	23.2 (19, 28.1)	42156.9 (33008.8, 49624.6)	21.5 (16.9, 25.3)	−0.2 (−0.5,−0.0)
	Death	1523.7 (1226.7, 1914.6)	1.3 (1.1, 1.7)	2926.3 (2177.2, 3499.3)	1.5 (1.1, 1.8)	0.4 (0.1,0.7)
High-middle SDI	Incidence	905 (663.1, 1174.8)	1.5 (1.1, 1.9)	2,816 (2093.3, 3631.5)	1.5 (1.1, 1.9)	−0.0 (−0.1,0.0)
	Prevalence	3735.3 (2677.8, 4970.5)	6.2 (4.4, 8.2)	12145.2 (8987.9, 15940.7)	6.5 (4.8, 8.5)	0.1 (−0.0,0.2)
	DALYs	28213.1 (17951.3, 43,607)	46.7 (29.7, 72.1)	33627.8 (21538.9, 50689.2)	17.9 (11.4, 26.9)	−2.7 (−2.9,−2.5)
	Death	1488.2 (926.8, 2354.8)	2.5 (1.5, 3.9)	2014.9 (1243.3, 3023.7)	1.1 (0.7, 1.6)	−2.3 (−2.5,−2.1)
Middle SDI	Incidence	447.7 (328.7, 584.2)	1.5 (1.1, 2)	1245.5 (919.2, 1612.2)	1.5 (1.1, 2)	−0.1 (−0.2,−0.1)
	Prevalence	1642.4 (1176.3, 2,177)	5.7 (4.1, 7.5)	4439.5 (3208.2, 5920.7)	5.5 (4, 7.3)	−0.1 (−0.2,0.0)
	DALYs	7490.9 (4711.1, 12957.1)	25.9 (16.3, 44.8)	12,196 (7768.2, 24,603)	15.1 (9.6, 30.5)	−1.5 (−1.6,−1.3)
	Death	369.6 (230.1, 656.9)	1.3 (0.8, 2.3)	644.1 (398.8, 1291.3)	0.8 (0.5, 1.6)	−1.2 (−1.4,−1.0)
Low-middle SDI	Incidence	547.3 (400.8, 714)	1.7 (1.3, 2.2)	1409.3 (1039.8, 1826.6)	1.7 (1.2, 2.2)	−0.1 (−0.1,−0.1)
	Prevalence	1765.5 (1286.1, 2328.8)	5.5 (4, 7.3)	5,022 (3677.9, 6,557)	6 (4.4, 7.9)	0.3 (0.3,0.4)
	DALYs	10204.4 (5361.6, 15933.6)	31.9 (16.8, 49.8)	17374.3 (8574.7, 32391.7)	20.8 (10.3, 38.8)	−1.2 (−1.3,−1.1)
	Death	481.7 (249.7, 764.3)	1.5 (0.8, 2.4)	902 (421.2, 1749.5)	1.1 (0.5, 2.1)	−0.9 (−1.0,−0.8)
Low SDI	Incidence	594.4 (434.7, 773.5)	2 (1.5, 2.6)	1,449 (1072.8, 1885.2)	1.9 (1.4, 2.4)	−0.3 (−0.3,−0.2)
	Prevalence	1746.1 (1266.9, 2292.6)	5.9 (4.3, 7.7)	4510.8 (3327.9, 5924.8)	5.8 (4.3, 7.7)	−0.1 (−0.1,−0.0)
	DALYs	10705.9 (5313.7, 17220.2)	36.1 (17.9, 58.1)	23510.4 (10979.1, 42218.5)	30.5 (14.2, 54.7)	−0.5 (−0.6,−0.4)
	Death	508.7 (245.9, 812.5)	1.7 (0.8, 2.7)	1,178 (526, 2209.2)	1.5 (0.7, 2.9)	−0.3 (−0.4,−0.3)
China	Incidence	1042.4 (774.2, 1379.2)	1.4 (1.1, 1.9)	3057.7 (2284.7, 3938.9)	1.4 (1.1, 1.8)	−0.1 (−0.2,−0.1)
	Prevalence	4244.3 (3086.4, 5656.1)	5.8 (4.2, 7.8)	12724.7 (9350.4, 16764.8)	6 (4.4, 7.9)	0.2 (0.1,0.3)
	DALYs	24764.6 (15437.5, 40947.1)	34 (21.2, 56.2)	38644.7 (23342.2, 60279.7)	18.1 (10.9, 28.2)	−0.9 (−1.3,−0.5)
	Death	1311.9 (817.3, 2227.2)	1.8 (1.1, 3.1)	2453.5 (1,498, 3807.4)	1.1 (0.7, 1.8)	−0.4 (−0.8,0.0)

### Joinpoint trends in ASMR among women aged ≥55 years

**Figure 6:**
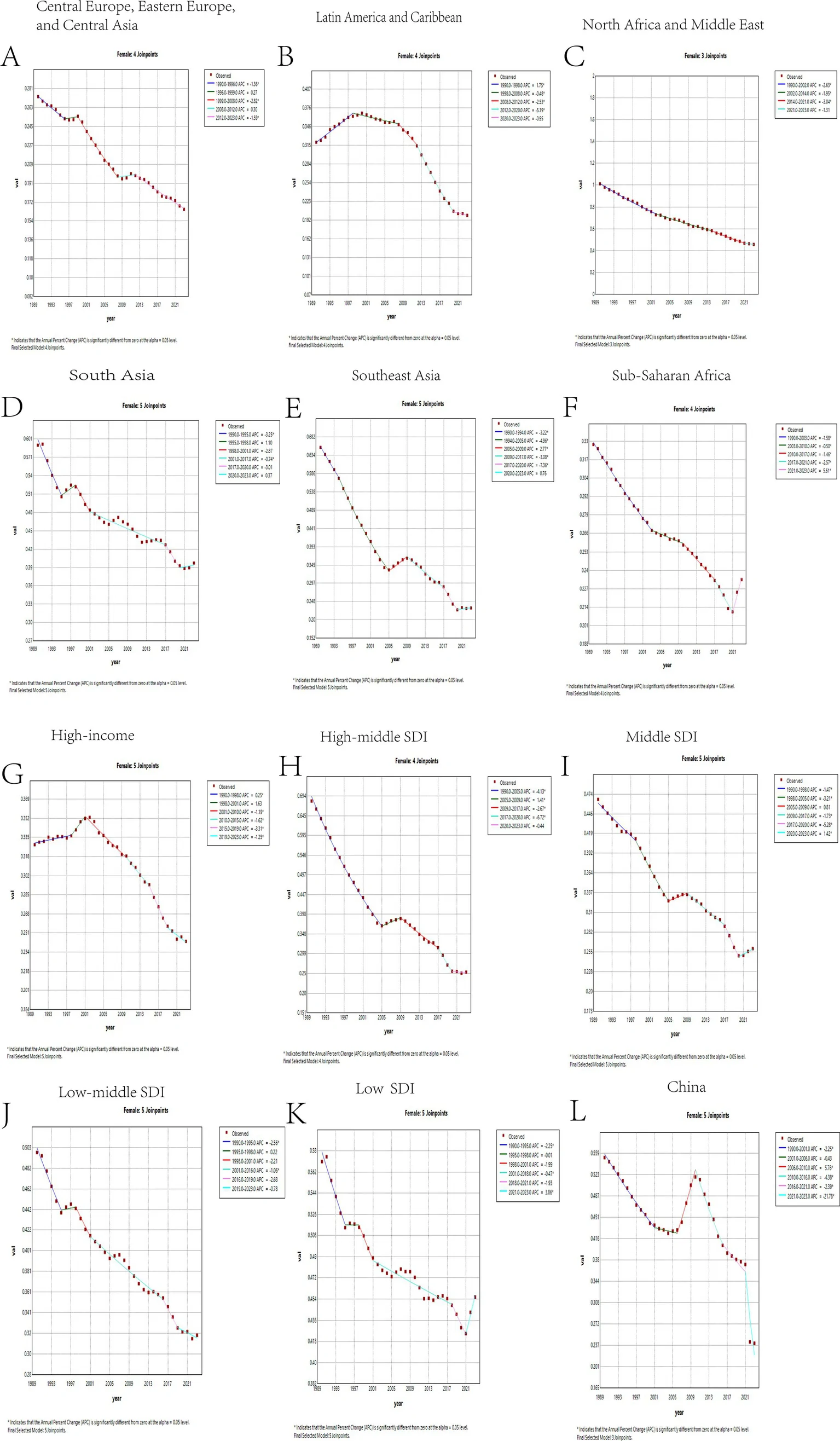
**(A–L)** Depict Joinpoint regression trends for ASMR in women aged ≥55 years across 12 global regions from 1990–2023. APC, annual percentage change.

From 1990–2023, joinpoint analysis showed that the ASMR of women aged ≥55 years with pulmonary hypertension went down a lot around the world. However, the size and timing of the drop were very different in different parts of the world. Sub-Saharan Africa and low-SDI regions saw the biggest drops in ASMR, from about 1.00–1.10 to 0.25–0.30. Between 1995 and 2005, there were 1–2 major inflection points that showed the rate of decline sped up at different stages. South Asia and Southeast Asia both saw big drops, from about 0.60 to 0.70 to about 0.18 to 0.20. But Latin America, the Caribbean, North Africa, and the Middle East all saw steady, straight drops that came together to about 0.15 to 0.18 by 2023. Central Europe, Eastern Europe, and Central Asia all went down from about 0.55 to about 0.20 without any big changes. High-income and high-middle SDI areas had the lowest death rates throughout the study period. Death rates dropped slowly from about 0.30 to about 0.10, with joinpoints showing a slow drop in the early years followed by a faster drop after 2005. China had a unique, non-linear, three-phase pattern: a slow fall from 1990 to 2005, a short-lived rise peaking near 0.33 around 2015, and then a sharp drop to 0.12 by 2023. This made China stand out from all the other regions. Overall, most regions had significantly negative APC values and negative AAPC estimates. This confirmed that the death rate for women aged ≥55 years with pulmonary hypertension has been going down for a long time, though the rate and size of improvement varied a lot between regions and levels of development (Figure 6).

### Decomposition of factors driving the global burden of PAH

**Figure 7:**
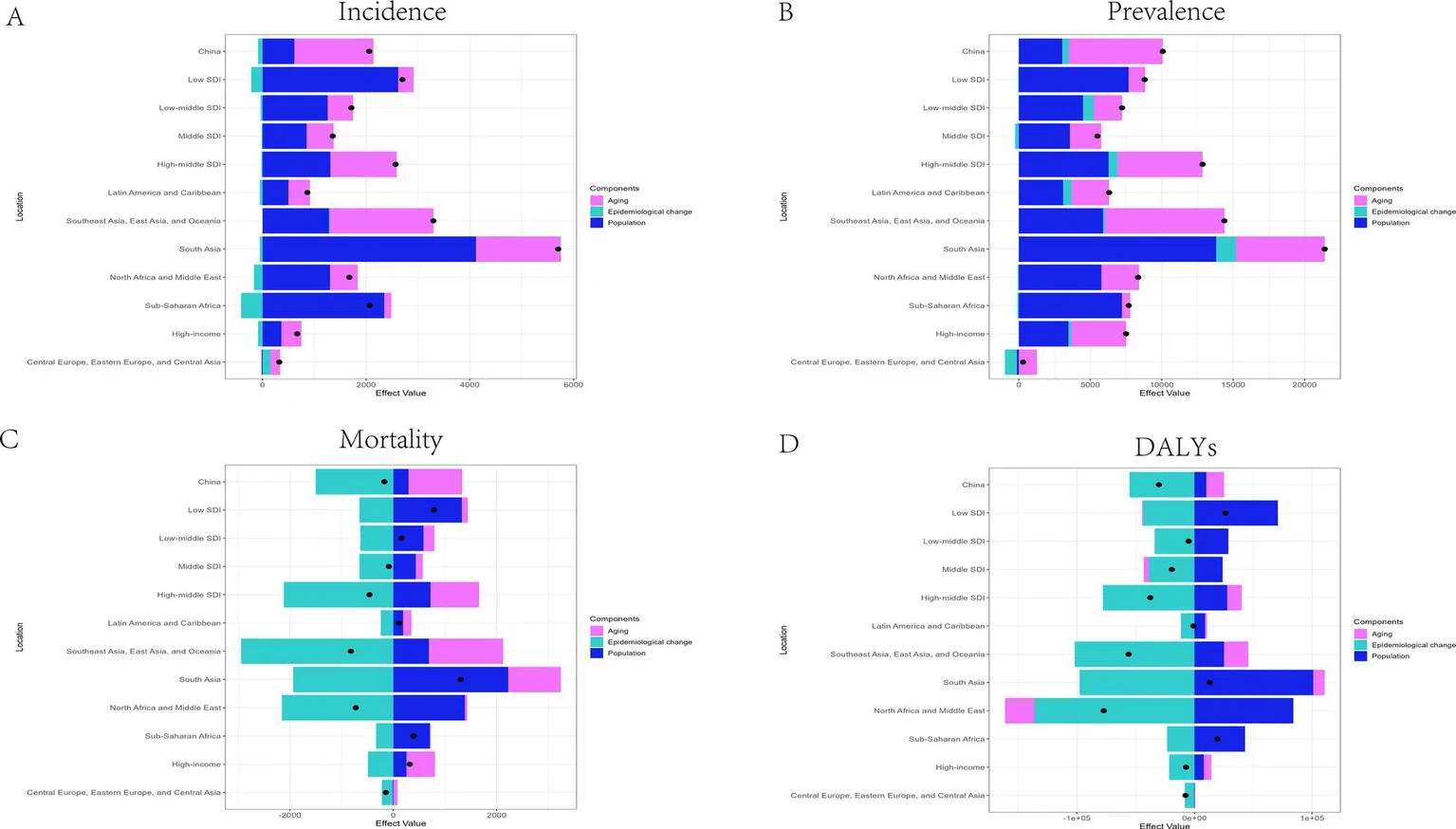
Decomposition analysis of the contributions of aging, epidemiological changes and population factors to the incidence rate **(A)**, prevalence rate **(B)**, mortality rate **(C)**, and disability-adjusted life years rate **(D)** of PAH in women. Positive values on the x-axis indicate an increase in incidence or mortality, while negative values indicate a decrease. The black dots represent the total impact of these three factors.

The decomposition analysis revealed that three factors—population expansion, population aging, and epidemiological change—were the primary contributors to the changes in the burden of PAH among women from 1990–2023 (Figure 7). Population growth was the primary factor contributing to increases in incidence and prevalence, especially in South Asia, Southeast Asia–East Asia–Oceania, and sub-Saharan Africa, where rapidly growing female populations resulted in significant rises in absolute cases and DALYs, despite declines in age-standardized rates. The aging population significantly contributed to the rise in mortality and DALYs in high-income regions, China, and high-middle SDI countries, in line with the increasing percentage of older women. Aging exerted a far lesser influence on sub-Saharan Africa and several regions of South Asia owing to their youthful demographic compositions. Epidemiological changes exhibited distinct regional divergence: negative in high-income regions, China, and certain areas of Latin America, signifying decreases in underlying disease risk, while strongly positive in sub-Saharan Africa, South Asia, and the Middle East–North Africa, reinforcing upward trends across all indicators. In summary, population growth primarily elevated case numbers, aging disproportionately intensified mortality and DALYs, and epidemiological shifts influenced the extent and nature of regional disparities.

### Cross-national PAH health inequality

**Figure 8:**
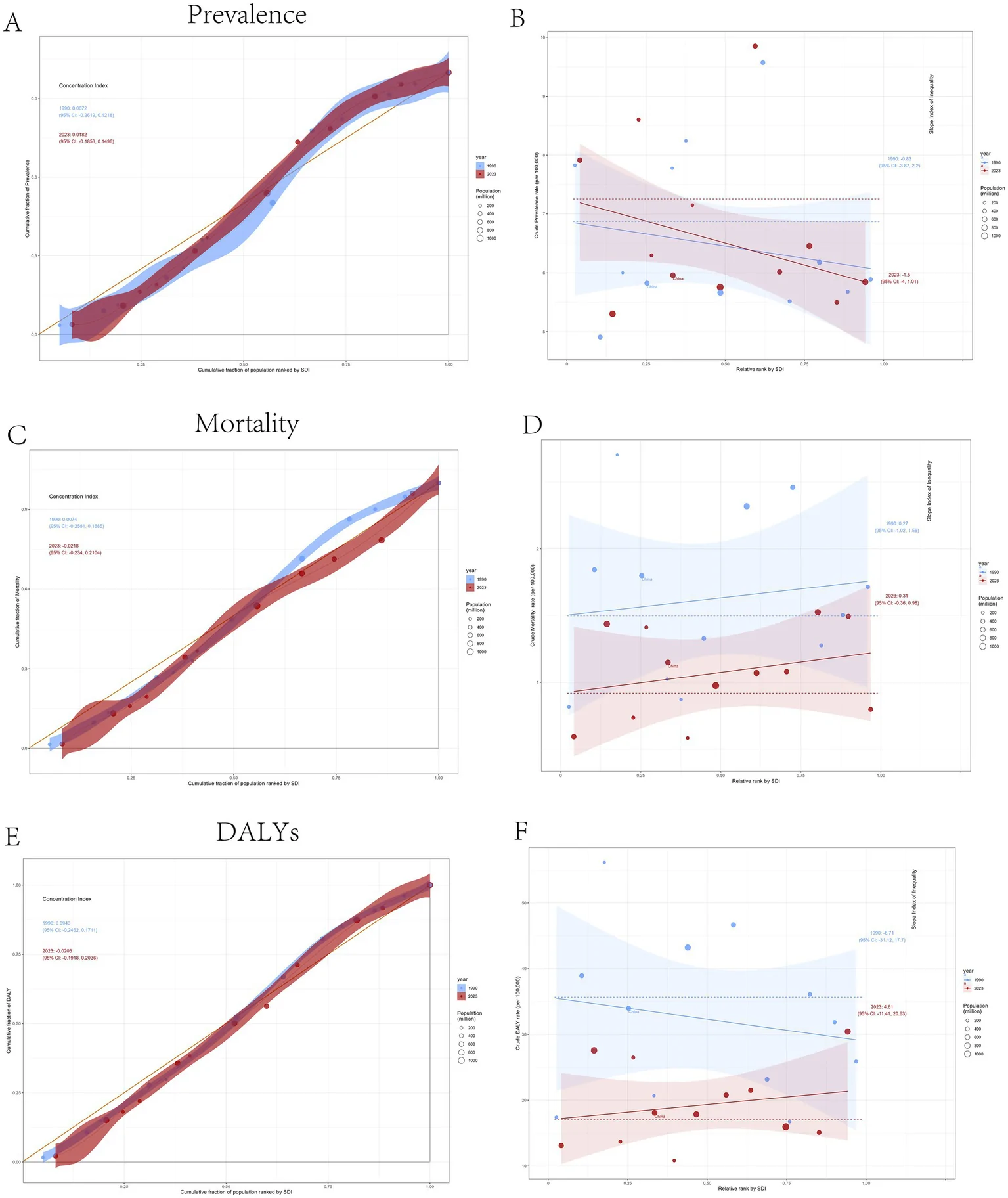
Health inequality assessment of PAH-related prevalence **(A,B)**, mortality **(C,D)**, DALYs **(E,F)** across SDI regions, 1990 vs. 2023.

The decomposition analysis utilizing the Concentration Index (CI) and Slope Index of Inequality (SII) indicates that from 1990–2023, a degree of inequality persists in the prevalence, incidence, mortality, and DALYs of PAH among women aged ≥55 years across various SDI regions; however, the overall disparity has demonstrated a gradual convergence trend (Figure 8). Regarding mortality rate, the SII in 1990 was 0.2705 (95% CI: −1.017 to 1.5581), increasing to 0.3112 in 2023 (95% CI: −0.3599 to 0.9822), indicating a marginal rise in mortality burden alongside the increase in SDI, although the difference was not statistically significant. The CI diminished from 0.0074 in 1990 (95% CI: −0.2581 to 0.1685) to −0.0218 in 2023 (95% CI: −0.234 to 0.2104), reflecting a minor negative shift that suggests a more equitable distribution of the mortality burden across various SDI regions. Regarding DALYs, CI experienced a marginal decline from 0.0943 in 1990 (95% CI: −0.2462 to 0.1711) to −0.0203 in 2023 (95% CI: −0.1918 to 0.2036), indicating a decrease in the disparity of health loss burden. In summary, both CI and SII exhibit a relatively low degree of inequality across all measures, with a significant reduction observed from 1990–2023. Despite minor disparities among various SDI regions, the global disease burden of pulmonary hypertension in women aged ≥55 years regarding socio-economic factors is progressively converging.

### Hierarchical clustering analysis

**Figure 9:**
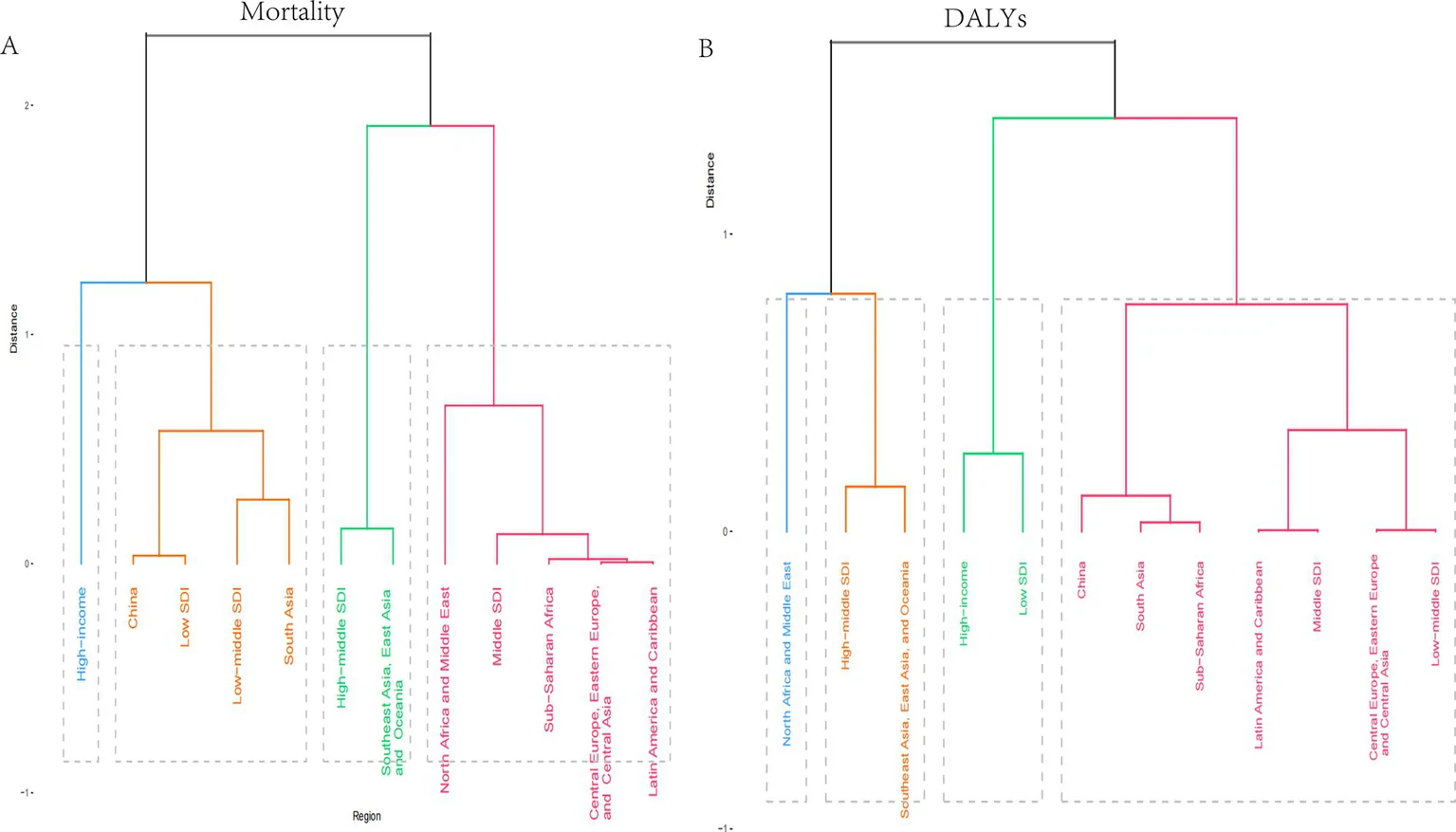
Hierarchical clustering of EAPC for mortality rate **(A)** and DALYs **(B)** in women aged ≥55 years with PAH from 1990–2023.

Hierarchical clustering of regional EAPC values for mortality rates and DALYs in women aged ≥55 years with pulmonary hypertension identified four distinct evolutionary patterns (Figure 9; Supplementary Table S3). The clusters for mortality had significantly distinct EAPC centroids (−1.25, −0.56, 0.41, −2.38). Regions in Cluster 1—Central Europe, Eastern Europe and Central Asia, Latin America and the Caribbean, Middle SDI, North Africa and the Middle East, and Sub-Saharan Africa—exhibited moderate decreases. Cluster 2 (China, Low-SDI, Low-middle SDI, and South Asia) demonstrated less pronounced reductions. High-income regions constituted a distinct cluster with upward trends, whereas the High-middle SDI, along with Southeast/East Asia and Oceania, aggregated into the cluster demonstrating the most pronounced fall. A similar four-cluster configuration was identified for DALYs, featuring EAPC centroids of −1.16, −0.38, −2.76, and −2.07. Most middle-SDI and Asian regions were categorized into the moderate-decline group, whereas high-income and low-SDI locations were grouped into the mild-decline category. The most rapid reductions in DALY were noted in High-middle SDI regions and Southeast/East Asia and Oceania, while North Africa and the Middle East had a unique pattern of significant decline. Both indicators collectively exhibited a consistent gradient, transitioning from a gradual fall to a steep decline, beside one group showing an increasing trend, underscoring significant regional variability in long-term burden trajectories.

### Frontier analysis

**Figure 10:**
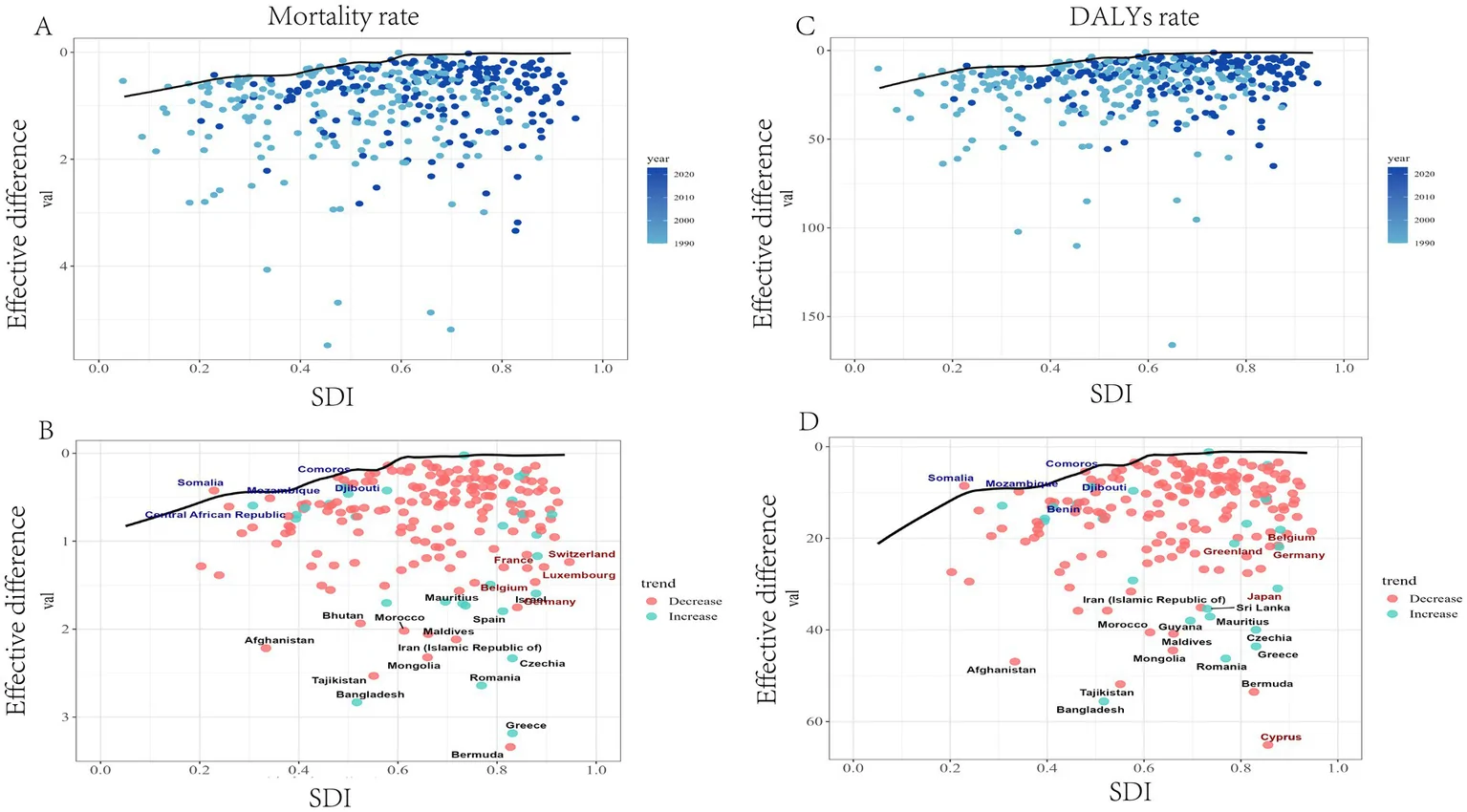
Frontier analysis exploring the relationship between the SDI and the mortality rate or DALYs. The solid black curve represents the frontier line, indicating countries or territories with exemplary performance that achieved the lowest mortality or DALYs burden relative to their SDI. Each dot corresponds to a specific country or region. In **(A,C)**, the color gradient represents the calendar years from 1990 (lighter shades) to 2023 (darker shades). In **(B,D)**, the top 15 countries and territories with the largest effective difference (i.e., the largest gap in mortality or DALYs burden from the frontier) are labeled in black. The direction of burden change from 1990–2023 is indicated by dot color, with red representing a decrease and blue representing an increase.

Frontier analysis based on Data Envelopment Analysis (DEA) demonstrated a nonlinear negative relationship between SDI and the age-standardized mortality rate (ASMR) and age-standardized DALY rate (ASDR) among women aged ≥55 years (Figure 10). Both indicators declined with increasing SDI and approached a plateau at higher SDI levels (approximately 0.8–1.0). From 1990 to 2020, most countries shifted downward toward the frontier, indicating overall reductions in mortality and DALY burden, although substantial variation in the distance to the frontier persisted across SDI levels. Several low-SDI countries, including Somalia, Mozambique, Comoros, Chad, and the Central African Republic, were located close to the frontier, whereas a number of middle- and high-SDI countries, including France, Switzerland, Luxembourg, Germany, Spain, and Belgium, remained farther from the frontier, indicating higher burden than expected for their level of socio-demographic development. Similarly, several middle-SDI countries, such as Iran, India, Bangladesh, and Tajikistan, also exhibited relatively large distances from the frontier.

### Projections of the global trends until 2040

**Figure 11:**
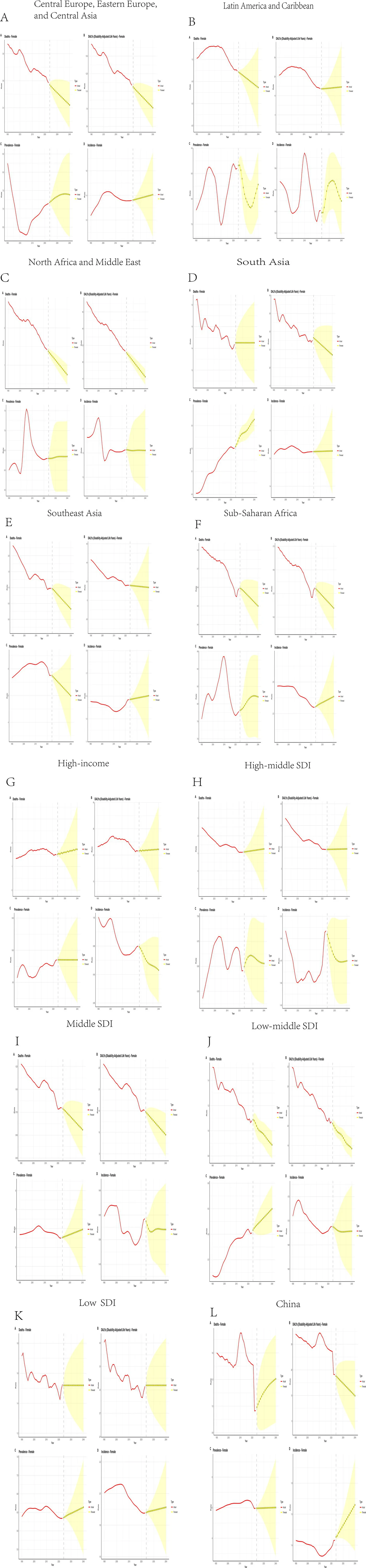
ARIMA forecast results for ASIR, ASPR, ASMR, and ASDR in women aged 55 and above across different regions from 1990 to 2040. The red line represents historical estimates, the yellow band indicates the forecast interval (95% CI), and the dashed line shows the projected trend.

The ARIMA model’s prediction of age-standardized indicators for women aged ≥55 years with PAH from 1990–2023 shows that while future trends are generally stable across regions, there are clear regional differences in magnitude (Figure 11). Overall, ASIR and ASPR remain stable or show a slight increase in most regions (predictions mostly fall within the range of 0.8–1.5/10^5^ and 5–8/10^5^), while ASMR and ASDR continue to decline globally, with predicted ranges generally between 0.1–0.6/10^5^ and 10–30/10^5^. In regions such as high SDI countries, East Asia–Southeast Asia–Oceania, Central Europe–Eastern Europe–Central Asia, Latin America, and North Africa–Middle East, ASMR and ASDR have shown significant declines over the past 30 years—mostly from around 0.4–0.8/10^5^ (ASMR) and 20–30/10^5^ (ASDR) in 1990, to 0.1–0.3/10^5^ and 10–18/10^5^ in 2023. Predictions indicate that these regions will continue to experience a gradual decline in the future with a moderate slope. In high-SDI regions, all four indicators are at the lowest levels globally. The heavily burdened South Asia and Sub-Saharan Africa are expected to maintain relatively high levels during the forecast period, with ASMR generally in the range of 0.4–0.6/10^5^ and ASDR between 20–30/10^5^. The forecast trends for China exhibit distinct characteristics (Figure 11L): ASIR has consistently risen during the historical period, increasing from approximately 1.1/10^5^ in 1990 to about 1.4/10^5^ in 2023; indicating a potential future plateau at high levels of around 1.4–1.6/10^5^. ASMR and ASDR have rapidly declined since 2010, dropping to about 0.15–0.2/10^5^ (ASMR) and 15–18/10^5^ (ASDR) by 2023. The results suggest a continued decline in the future, with ASMR possibly dropping further to ~0.1/10^5^, ASDR decreasing to 12–15/10^5^, and ASPR remaining stable throughout the forecast period, mostly falling within the range of 5–6/10^5^, with no significant fluctuations. The reliability of these projections is supported by backtesting results (Supplementary Table S4), where all indicators achieved MAPE < 20% (meeting the pre-defined accuracy threshold). Readers are advised to refer to Supplementary Table S5 for detailed annual performance and Supplementary Supplementary Figure S1 for intuitive metric comparisons.

## Conclusion

This study reveals significant and enduring geographical disparities in the prevalence of PAH among women aged 55 years and older worldwide. Individuals aged 55 to 69 represent a critical demographic for the rapid onset of PAH risk and warrant prioritization for preventative strategies. High and middle SDI countries must swiftly establish long-term monitoring and chronic management systems specifically designed for women aged ≥55 years. Targeted policies and healthcare services are crucial for mitigating the global impact of PAH.

## Discussion

This study utilizes GBD 1990–2023 data to deliver a thorough evaluation of the global, regional, and national burden of PAH among women aged ≥55 years. Multiple significant findings were revealed. Initially, all four age-standardized indicators exhibited significant regional variability, with Central Asia and sub-Saharan Africa representing high-burden clusters, whilst high-income countries consistently recorded the lowest values. The correlation between PAH burden and SDI exhibited a nonlinear, multidimensional pattern: ASIR and ASDR decreased with rising SDI, whereas ASPR peaked in middle- to high-SDI nations, indicating a growing population affected by chronic illness. Third, between 1990–2023, ASMR and ASDR diminished in most locations, although the extent and timing of the declines varied significantly; China had a unique three-phase pattern characterized by a gradual fall, a brief surge, and a swift reduction. Fourth, among persons aged ≥55 years, women exhibited consistently higher ASIR and ASPR than men—frequently 1.3 to 1.8 times greater—whereas sex disparities in ASMR and DALYs were comparatively minor, signifying a greater prevalence of disease in women but similar mortality risk. Fifth, decomposition analysis revealed that population growth and aging were the primary factors contributing to the increase in absolute burden, while epidemiological changes varied among regions and predominantly influenced interregional disparities. Ultimately, global disparities in PAH burden among women aged ≥55 years remained minimal and continued to diminish; however, discrepancies in health-system performance persisted: several low-SDI countries attained burden levels near their anticipated frontier, while certain high-SDI countries exhibited suboptimal outcomes despite superior resources. The large cross-national differences in ASMR should be interpreted with caution, as extreme values in countries with limited primary health data may be influenced by GBD model-based estimation and data sparsity, particularly in low-SDI settings. Nevertheless, the overall pattern of higher burden in Central Asia and sub-Saharan Africa remained consistent across multiple indicators.

PAH is regarded as one of the most progressive and life-threatening cardiovascular disorders, marked by irreversible vascular remodeling, elevated death rates, and significant functional impairment (24). It is enveloped in despair, diminishes quality of life, has a high death rate, and imposes a considerable weight on families, society, and nations (25, 26). The pronounced spatial clustering of PAH among older women in Central Asia and sub-Saharan Africa corresponds with the overarching trend of cardiovascular and respiratory illness prevalence in low-income regions. In these areas, multiple structural factors collectively increase risk: postponed diagnosis, restricted access to right heart catheterization—the diagnostic gold standard—high incidence of advanced pulmonary hypertension complications, and the ongoing prevalence of infections known to induce or exacerbate pulmonary vascular disease, such as HIV, schistosomiasis, and tuberculosis (27–30). Environmental and sociodemographic factors, such as biomass fuel burning, household air pollution, anemia, and chronic inflammatory conditions, disproportionately impact women and may exacerbate age- and hormone-related susceptibility (31, 32). Conversely, affluent locations exhibit comparatively low mortality and DALYs despite elevated prevalence, indicative of efficient early identification, established public health referral networks, and extensive access to targeted medicines. These systems facilitate earlier diagnosis, enhanced monitoring, and improved long-term survival, resulting in a disease profile defined by “higher prevalence but lower mortality”—a significant contrast to the “high incidence and high mortality” pattern observed in low SDI settings. Consistently high ASMR and ASDR in certain regions of Eastern Europe and Central Asia probably indicate increased cardiovascular risk, postponed diagnoses, obstacles to specialist care, and disjointed chronic illness management systems.

This study identifies a clear susceptibility window for PAH in women aged ≥55 years, with incidence peaking at 55–59 years, coinciding with the hormonal transition of menopause. Several biological mechanisms have been proposed to explain the increased susceptibility to pulmonary vascular disease after menopause. Previous experimental and clinical studies suggest that declining estrogen levels may be associated with reduced endothelial nitric oxide signaling, altered prostacyclin pathways, and impaired anti-inflammatory regulation, potentially contributing to pulmonary vascular remodeling (33, 34, 35). When superimposed on long-term exposure to hypertension, metabolic syndrome (36), and chronic inflammation, these changes may push susceptible women beyond the threshold for pulmonary vascular injury. Moreover, women bear a lifelong burden of autoimmune diseases—particularly systemic sclerosis and systemic lupus erythematosus—major contributors to connective tissue disease–associated PAH (CTD–PAH) (37). Many of these diseases become clinically apparent or accelerate in midlife, coinciding with menopause. Importantly, estrogen’s biological effects in PAH are not uniformly protective. Depending on receptor subtype (ERα, ERβ, GPR30), metabolite profile, and local tissue environment, estrogens may protect the right ventricle while simultaneously promoting pulmonary vascular proliferation and altering BMPR2 signaling (38). Menopause-related hormonal alterations, including reductions in estradiol and DHEA-S, have been hypothesized to influence pulmonary vascular susceptibility in predisposed individuals (39). Despite higher incidence and prevalence, mortality differences between women and men remain modest. This aligns with the “estrogen paradox,” in which women’s right ventricular (RV) function demonstrates superior adaptation under pressure overload, with better contractile reserve, cardiopulmonary coupling, and metabolic efficiency. However, this apparent functional advantage does not translate uniformly into improved survival across all PAH subtypes, as clinical outcomes are strongly influenced by underlying etiology, comorbid conditions, and stage at diagnosis (38, 40, 41). Superior right ventricular adaptation observed in previous clinical studies may partially contribute to the relatively modest sex differences in mortality observed in population-level data, although survival differences between sexes vary substantially across PAH etiologies and are not consistently in favor of women in all clinical subgroups. Importantly, the present study is based on ecological estimates from the GBD database and cannot directly verify the biological mechanisms underlying the observed sex- and age-related differences. Therefore, the mechanistic interpretations discussed above should be considered hypothesis-generating and supported primarily by previous experimental and clinical literature rather than direct causal inferences from the current analysis. Overall, while female sex may confer relative advantages in right ventricular adaptation, the net prognostic effect is heterogeneous and critically dependent on disease etiology, clinical phenotype, and healthcare access.

The regional heterogeneity in the burden of PAH among older women in 2023 is largely shaped by three interacting forces: population growth, population aging, and evolving epidemiological risks (42). In South Asia, Southeast Asia, and sub-Saharan Africa, rapid expansion of the female population combined with long-term exposure to high-risk environments—such as biomass smoke, air pollution, anemia, and chronic infections—has driven a continued rise in absolute cases despite declines in age-standardized rates. In these regions, the pace of population and risk expansion has exceeded the capacity of local health systems to effectively reduce the burden. In contrast, high-SDI countries show declining epidemiological risks due to improvements in CTD management, broader use of targeted therapies, and higher diagnostic coverage. However, rapid population aging means that increasing numbers of women aged ≥70 are entering the period of accelerated PAH-related functional decline and mortality, limiting further reductions in ASMR and DALYs despite advanced medical technologies (43). The challenge for these settings is not solely therapeutic advancement but converting available resources into population-level gains through integrated, efficient care models (44). China illustrates a mixed pattern. As one of the fastest-aging nations, aging has become the dominant structural driver of mortality and DALYs. At the same time, substantial improvements in air quality (45), standardized CTD–PAH management, and expanded access to targeted therapies (46) have markedly reduced epidemiological risks, producing the sharp declines in ASMR and ASDR observed since 2015. Nonetheless, persistent PM₂.₅ exposure (47), a high baseline prevalence of autoimmune diseases among women, and urban–rural disparities in healthcare limit China’s frontier performance relative to countries with similar SDI levels. Although national screening programs, improved referral networks, and specialized PH platforms have strengthened the overall PAH care system (48), targeted strategies for menopausal women remain insufficient (49). Given that menopause represents a critical window for PAH onset, women aged 55–59 should be prioritized for early identification, community-based screening, and structured risk assessment. Cross-disciplinary early screening pathways for high-risk subtypes—such as CTD-PAH, HIV-PH, and high-altitude–related PH—linking gynecology, rheumatology, and cardiopulmonary care may facilitate diagnosis before symptom onset, enhance pre-RHC evaluation efficiency, and reduce diagnostic delay.

The mechanistic interpretations discussed in this study should be interpreted with caution. Although previous experimental and clinical studies have suggested potential roles of estrogen signaling, vascular remodeling, and immune-inflammatory pathways in PAH, the present analysis is based on ecological GBD estimates rather than individual-level clinical data. Consequently, the observed differences should be interpreted as population-level associations and temporal patterns rather than evidence of causal or biological mechanisms. In addition, women aged ≥55 years were used as a population-level proxy for postmenopausal status, and menopausal status itself was not directly measured. Therefore, the findings should not be interpreted as demonstrating effects attributable specifically to menopause or estrogen withdrawal.

This study possesses various limitations that necessitate careful interpretation. The analysis relies on estimations from the GBD model, and many low SDI countries possess insufficient raw data, resulting in subpar disease coding quality. Secondly, the categorization of PAH in the GBD lacks complete alignment with the clinical WHO Group 1 PAH, complicating the precise distinction among idiopathic, inherited, and secondary PAH. This study primarily addresses the ‘burden of pulmonary vascular disorders linked with PAH,’ indicating potential classification overlap. This study concentrates on individuals aged ≥55, approximating the postmenopausal demographic, while excluding critical individual-level data such as age at menopause, hormone replacement medication, and comorbidities. The data primarily indicate population-level correlations. Although women aged ≥55 years are frequently used as a surrogate for postmenopausal status in population-level analyses, this approach cannot fully account for regional and ethnic heterogeneity in menopause timing. Consequently, some degree of exposure misclassification may exist, particularly in populations with earlier or later menopausal transition. Fourth, we excluded other ‘clinical’ variables such as treatment accessibility, utilization of specific drugs, or rates of right cardiac catheterization, hence constraining the examination of specific intervention routes underlying the ‘epidemiological consequences’. Although the ARIMA model showed acceptable fitting performance for historical trends, caution is warranted when interpreting forecasts extending to 2040. ARIMA models are primarily optimized for short- to medium-term prediction and may become less reliable over longer forecasting horizons. The widening prediction intervals in our analysis reflect increasing uncertainty over time. Additionally, ARIMA projections cannot fully account for future changes in demographics, healthcare systems, therapeutic advances, or public health interventions that may influence pulmonary hypertension burden trends. Notably, backtesting results (Supplementary Table S4) show that ASIR and ASPR had a 95% prediction interval coverage rate of only 12.5%—a finding potentially attributed to unforeseen shifts in PAH diagnostic patterns post-2019 (e.g., changes in clinical screening protocols or healthcare access). This limitation is further illustrated in Supplementary Supplementary Figure S1, which visually highlights the gap between predicted intervals and actual values for ASIR/ASPR. Corresponding cautious interpretations of their long-term trends are also emphasized in the conclusion. Therefore, these projections should be interpreted as exploratory forecasts rather than precise long-term predictions. Future research must more effectively integrate global burden assessments with clinical registries and mechanistic studies: specialized PAH among older women registries could be established in typical SDI countries, while translational research pathways could be developed concerning menopausal hormone fluctuations, right ventricular remodeling, and microvascular lesions, thereby converting the macro patterns identified in this study into actionable, precise intervention strategies.

## Data Availability

The original contributions presented in the study are included in the article/Supplementary Supplementary material, further inquiries can be directed to the corresponding authors.

## Supplementary material

SUPPLEMENTARY TABLE S1 Age-standardized PAH burden indicators (ASIR, ASPR, ASMR, ASDR) among women aged ≥55 years across regions, 2023.

SUPPLEMENTARY TABLE S2 Baseline burden of PAH in women aged ≥55 years across regions, 1990–2023.

SUPPLEMENTARY TABLE S3 Hierarchical clustering of regional EAPC for PAH mortality and DALYs in women aged ≥55 years.

SUPPLEMENTARY TABLE S4 Backtesting validation metrics of ARIMA model for PAH age-standardized indicators (ASIR, ASPR, ASMR, ASDR) in women aged ≥55 years, 2016–2023.

SUPPLEMENTARY TABLE S5 Year-by-year comparison of predicted vs. actual PAH age-standardized indicators (ASIR, ASPR, ASMR, ASDR) in women aged ≥55 years, 2016–2023.
